# Repeated measures of Heparin-binding protein (HBP) and procalcitonin during septic shock: biomarker kinetics and association with cardiovascular organ dysfunction

**DOI:** 10.1186/s40635-020-00338-8

**Published:** 2020-09-10

**Authors:** Jonas Tverring, Niklas Nielsen, Josef Dankiewicz, Adam Linder, Fredrik Kahn, Per Åkesson

**Affiliations:** 1 Division of Infection Medicine, Department of Clinical Sciences, Lund University, SE-221 84 Lund, Sweden; 2 Department of Infectious Diseases, Helsingborg General Hospital, Helsingborg, Sweden; 3 Department of Anaesthesia and Intensive Care, Intensive Care Unit, Helsingborg General Hospital, Helsingborg, Sweden; 4 Division of Anaesthesiology and Intensive Care, Department of Clinical Sciences, Lund University, Lund, Sweden; 5 Department of Clinical Sciences, Division of Cardiology, Lund University, Lund, Sweden; 6 Department of Cardiology, Skåne University Hospital, Lund, Sweden; 7 Department of Infectious Diseases, Skåne University Hospital, Lund, Sweden

**Keywords:** Heparin-binding protein, Procalcitonin, Repeated measures, Kinetics, Sepsis, Septic shock, Phenotype

## Abstract

**Background:**

Heparin-binding protein (HBP) is a neutrophil-derived pro-inflammatory protein, an inducer of endothelial dysfunction and vascular permeability and a promising prognostic biomarker in sepsis. This exploratory study aims to describe the kinetics of plasma HBP during septic shock and investigate an association between repeated measures of HBP concentration and cardiovascular organ dysfunction severity.

**Methods:**

We included patients at or above 18 years with suspected septic shock on admission to the intensive care unit (ICU) during 2014 and 2016 to 2018. Plasma samples were collected from ICU admission and every 4 h for 72 h or until death or ICU discharge and batch analysed for HBP. Mean arterial blood pressure (MAP) and noradrenaline dose (NA dose) were recorded at each sampling time point, and systemic vascular resistance index (SVRI) was recorded when available from non-invasive monitoring. The association between HBP, NA dose, MAP and SVRI was assessed respectively using mixed-effects linear regression models. Procalcitonin (PCT) was used as a comparator.

**Results:**

A total of 24 patients were included. The kinetics of plasma HBP was highly variable over time, with occasional >2-fold increases and decreases in between 4-h measurements. Every 100 ng/mL increase in HBP corresponded to a 30% increase in NA dose in a crude model (95% CI 3 to 60%, *p* = 0.03, *n*_obs_ = 340), a 1.4-mmHg decrease in MAP in an adjusted model (95% CI − 1 to − 2.3 mmHg, *p* = 0.04) or a 99 dyne s cm^−5^ m^−2^ decrease in SVRI in another adjusted model (95% CI − 36 to − 162, *p* = 0.002, *n*_pat_ = 13). PCT had a stronger association to NA dose than HBP in a crude model but was not significantly associated to NA dose, MAP or SVRI in any time-adjusted model.

**Conclusions:**

Plasma HBP displayed a highly variable kinetic pattern during septic shock and was significantly associated to cardiovascular organ dysfunction severity over time.

## Background

Septic shock is a serious medical condition caused by a dysregulated inflammatory response to a severe infection leading to endothelial and cardiac dysfunction [1]. Heparin-binding protein (HBP) is a neutrophil-derived, pro-inflammatory protein that has a potentially central role in sepsis-related endothelial dysfunction [2]. Pre-produced HBP is rapidly released from secretory vesicles in response to infectious stimuli [3]. HBP induces neutrophil adhesion to the endothelial surface, rearrangement of the cytoskeleton, cell contraction and mitochondrial dysfunction in the endothelium [4–7]. This enables neutrophil extravasation to the infected tissue but also leads to adverse vascular leak and endothelial dysfunction [8]. HBP has been established as a promising sepsis biomarker [2]. Several studies of single early measurements of HBP in plasma have reported high prognostic accuracy on development and outcome of sepsis and septic shock when concentrations were above 15–30 ng/mL [9–11]. Yet only one study of repeated measures of HBP have been published to date, reporting less than once daily sampling [12]. Serial measurement of inflammatory biomarkers is often used in clinical practice, and the added prognostic value of repeated measures has been shown for lactate and procalcitonin (PCT) [13, 14]. Nevertheless, the kinetics of HBP during a septic disease course are largely unknown. Based on HBP’s pathophysiological role in sepsis, it was our hypothesis that the level of HBP in plasma may be directly associated with infection-induced circulatory failure. Accordingly, we aimed to describe the kinetics of plasma HBP during septic shock using frequent sampling, and to investigate an association between plasma HBP concentration and measures of cardiovascular organ dysfunction severity over time.

## Methods

### Study design and setting

This was a dual-centre, observational, convenience sampling study conducted at two ICUs in southern Sweden. Skåne University Hospital in Lund is a university hospital with nine general ICU beds and Helsingborg Hospital is a teaching emergency hospital with seven ICU beds. Each hospital serves a population of approximately 250,000. Patient enrolment occurred during February 2014 (Lund) and September 2016 to February 2018 (Helsingborg). The Regional Ethical Review Board at Lund University approved the study design in the years 2014 and 2016 (Dnr 2014/741 and 2016/271).

### Patients

Patients were screened for eligibility on admission to the ICU by the attending intensivist. The inclusion criteria were [1] age at or above 18 years and [2] suspected septic shock. Suspicion of septic shock was defined as an acute increase in total Sequential Organ Failure Assessment (SOFA) score ≥ 2 points caused by a suspected infection and a need for vasopressors to maintain a mean arterial pressure (MAP) ≥ 65 mmHg despite adequate fluid resuscitation and a serum lactate level of ≥ 2 mmol/L. There were no exclusion criteria. Patients fulfilling the inclusion criteria were asked to participate in the study and to give their written informed consent, or if unable, their next-of-kin was asked permission.

### Sample collection and analyses

Plasma sample collection was started within 2 h from ICU admission and repeated every 4 h for 72 h, or until death or ICU discharge. Samples were drawn from an arterial line into 2.7-mL sodium citrate tubes. Samples were centrifuged, and the plasma was aliquoted and frozen to − 80 °C within 1 h from collection. The samples were sent on dry ice from Sweden to Scotland for batch analysis of HBP. Plasma HBP concentration was measured in Axis-Shield Diagnostics’ central laboratory (Dundee, GB) using their commercial HBP enzyme-linked immunosorbent assay (ELISA). Plasma sample haemolysis constitutes a potential error source to the HBP concentration because of potential concurrent lysis of neutrophils and subsequent HBP release. Sample haemolysis was analysed semi-quantitatively in a separate sample aliquot using a light absorption-based haemoglobin index assay at the Department of Clinical Chemistry at Skåne University Hospital, Lund, Sweden, in 2019. Haemolysis was considered significant if the haemolysis index was at or above 10 (corresponding to approximately 1 g of haemoglobin per litre).

### Data collection

Data on the time of sampling, MAP, and vasopressor and/or inotrope type(s) and dose(s) were collected at the time of plasma sampling by a trained ICU nurse using a study-specific case report form. All other data, such as demographics, medical history, lab test results, data from non-invasive monitoring, physiological parameters and survival, were collected retrospectively from electronic health records. All data collection was blinded to the biomarker test results.

### Variables

HBP, PCT and lactate were measured and MAP, heart rate, noradrenaline (NA) dose, dobutamine dose, levosimendan and vasopressin dose were recorded every 4 h for the first 72 h (i.e. maximum 18 measurements per patient). Simplified acute physiology score 3 (SAPS 3) was recorded on the first day of ICU admission and SOFA score was recorded once daily for a maximum of 7 days per patient. Systemic vascular resistance index (SVRI) and cardiac index (CI) was registered every 4 h if available in patients with a non-invasive monitor from either pulse contour cardiac output (PiCCO) or non-invasive cardiac output monitoring (NICOM). Comorbidity was defined and recorded according to Charlson comorbidity index (CCI) [15].

### Outcome measures and comparison

Repeated measures of HBP and PCT plasma concentration and serial levels of MAP and NA dose were the main outcome measures. NA dose and MAP represent continuous variables of cardiovascular organ dysfunction severity included in the current SOFA-based definition of septic shock [16]. SVRI was considered an exploratory outcome measure. We chose to compare the strength of the association between HBP and cardiovascular organ dysfunction to that of PCT, a well-studied biomarker in severe infections [17].

### Statistical analyses

The main statistical method used was multilevel mixed-effects linear regression models. When the dependent variable had a skewed distribution, we used generalized linear mixed-effects models with a gamma distribution and a random intercept at the patient level. When the dependent variable was normally distributed, we used a linear mixed-effects regression models with a random intercept. When time was included as a co-variate in the analysis, we also added a random slope for time and used an unstructured covariance matrix. Model assumptions for the linear regression analyses with a normally distributed dependent variable were checked using a residual-fitted plot, residual histogram and qq-normal plot. Assumptions for models with a gamma distribution were checked using Pearson residuals and a qgamma plot. Variance inflation factor (VIF) was used to check for multicollinearity. Model fit was calculated using Akaike information criterion (AIC) for models with a gamma distribution and the coefficient for determination (*R*^2^) for normally distributed models. Likelihood ratio tests were used to compare difference in fit between nested linear mixed-effects models. Spearman’s *R* was used to calculate correlation coefficients and *p* values for two continuous variables in the same patient as well as in summarized continuous variables. Wilcoxon rank-sum test was used to test for a significant difference between non-normally distributed continuous variables between two groups. The chi-square test was used to test for a significant difference in dichotomized variables between two groups. Missing observations were assumed to be missing at random (MAR) and were handled within the mixed-effects analysis. Results are presented with 95% confidence intervals (95% CI) and *p* values below 0.05 were regarded as statistically significant. Due to the exploratory nature of the study, no power calculation was performed. Statistical analyses were performed using Stata (Stata MP 16.1, StataCorp, Texas, USA). Ado-package multilevel tools (mlt) was used to calculate the coefficient for determination (*R*^2^) for linear mixed-effects models (Snijders and Bosker) (Katja Moehring and Alexander Schmidt, University of Cologne, 20130129). Ado-package quantile-quantile plot for data versus fitted gamma distribution (qgamma) was used to check assumptions for gamma distributed mixed-effects models (Nicholas J. Cox, University of Durham, 20110517). A do-file containing the Stata code for any result with a reported *p* value can be found in a supplementary file. This manuscript is reported following the STROBE statement [18]; see an attached checklist in a supplementary file.

## Results

### Patient characteristics

**Fig. 1:**
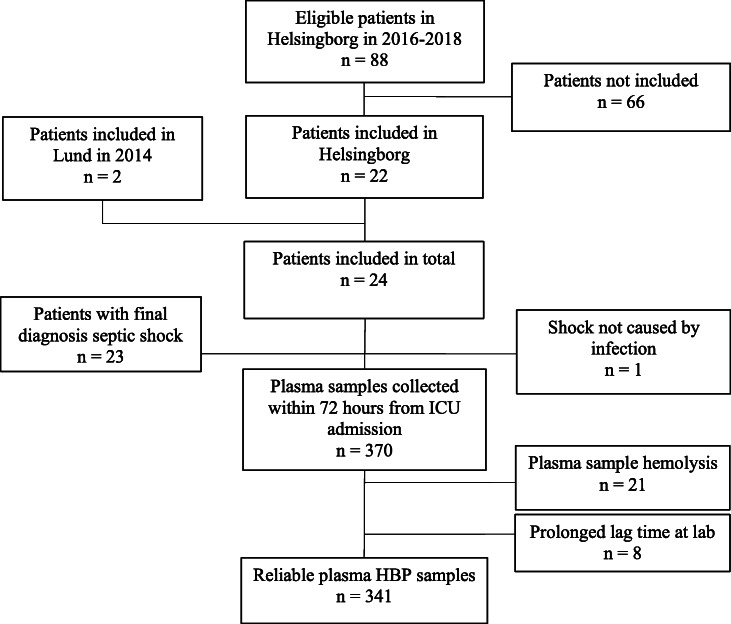
Flow chart of patients and samples

Twenty-four patients with vasopressor-dependent circulatory failure and suspected sepsis were included. The median age was 66 years (range 23 to 84), 11 patients (46%) were female and 8 patients (33%) were surgical admissions. At inclusion, the median SAPS 3 was 70 points (range 47–100), and the median SOFA score on day 1 was 11 (range 4–16). The final diagnosis was septic shock in 23 of the 24 patients. One patient (id 20) had circulatory failure from a non-infectious cause: a severe episode of inflammatory bowel disease. The 28-day mortality was 21% (5 of 24 patients) and the 90-day mortality was 25% (6 of 24). The median time to death was 9 days (range 1–72 days). For details on the patient characteristics, see Table 1. There were a total of 88 eligible patients during the major study period at Helsingborg Hospital in 2016 to 2018. Patients not included (*n* = 66) had a median age of 69 years, 47% were female, median SAPS 3 score was 72 points, 33% were surgical admissions and the 90-day all-cause mortality was 35%. The main reasons for not being included in the study despite eligibility were considered to be absence of a trained ICU study nurse at the time of admission, lack of incentive from the attending ICU physicians or lack of time due to a contemporary high workload. See flow chart in Fig. 1.

**Table 1:** Characteristics of the study population

ID	Age (years)	Sex (M/F)	Comorbidities	Type of infection (HAI or CAI)	Time from ED to ICU (hours)	Microbiology (blood or other)	Max SOFA score	Max lactate (mmol/L)	Days in ICU	Alive/dead within 90 days	No. of measurements	Spearman’s *R* (NA vs HBP)	*P* value
1	23	F	–	LRI	21	S aureus	12	3.9	23	Alive	15	0.73	< 0.01
2	69	F	–	Cholangitis	6	S anginosus, Prevotella spec., P micra	7	5.6	5	Alive	11	0.74	< 0.01
3	34	F	–	LRI	3	Negative	6	3.6	2	Alive	12	0.30	0.34
4	64	M	CKD, DM, CHF, TIA	IE	30	S aureus	13	3.3	9	Alive	18	0.11	0.68
5	80	M	Lymphoma	LRI	1	Actinomyces S anginosus	14	8.4	8	Alive	18	0.73	< 0.001
6	73	M	TIA	Cholangitis	3	E coli C perfringens	16	8.3	14	Alive	18	0.08	0.75
7	70	M	–	GI perf	5	Anaerobes	8	3.4	3	Alive	17	− 0.10	0.69
8	83	M	DM, MI	LRI	8	Influenza A (NP swab)	15	4.3	15	Dead [15]	18	0.06	0.82
9	75	F	DM	SSTI	9	S pyogenes	10	4.0	3	Alive	15	0.70	< 0.01
10	52	M	C1qD	Pharyngeal abscess	0	S pyogenes	18	22.0	2	Dead [2]	15	− 0.70	< 0.01
11	67	M	CKD, DM	Cholangitis (HAI)	563	E coli	16	3.8	7	Dead [7]	16	0.61	0.01
12	81	M	CHF	Unknown	1	Negative	11	11.6	2	Alive	17	− 0.02	0.94
13	57	F	COPD	UTI	2	E coli	13	5.3	3	Alive	13	0.56	< 0.05
14	77	M	AUD	LRI	6	Negative	15	8.0	1	Dead [1]	1	–	–
15	65	M	CKD	LRI	1	L pneumo (NP swab)	13	3.4	12	Dead [12]	16	0.48	0.06
16	63	M	CKD, CHF	LRI	0	H influenzae (NP swab)	8	2.5	1	Alive	17	− 0.37	0.15
17	65	F	CKD, DM	UTI	7	E coli	9	2.3	1	Alive	12	− 0.28	0.37
18	53	F	CLD	SSTI	1	Negative	11	5.2	2	Alive	9	0.10	0.80
19	84	F	COPD, CHF	SSTI	12	S dysgalactiae (wound swab)	12	3.4	3	Alive	3	− 0.87	0.33
20	66	F	TIA	No inf. (IBD)	2	Negative	9	3.5	2	Alive	17	0.03	0.91
21	45	M	DM	SSTI	34	S pyogenes	8	3.7	3	Alive	13	0.11	0.73
22	73	F	COPD, MM	LRI	0	Negative	15	5.3	11	Dead [11]	18	− 0.15	0.55
23	67	M	CKD	GI perf	6	K pneumoni E faecium	11	5.7	14	Alive	17	− 0.33	0.19
24	47	F	–	LRI	1	S pyogenes	15	11.0	11	Alive	15	0.62	0.01

Characteristics of patient population and results for individual plasma HBP to NA dose correlation over time. Comorbidities are reported as mentioned in the patient’s chart. Infections are community-acquired if not described as hospital-acquired (HAI). Microbiology is shown from blood culture if positive, and if negative, from any other positive culture site. Maximum SOFA and lactate are displayed within 72 h from ICU admission, respectively. Alive or dead is observed up to 90 days from ICU admission. The number of reliable HBP measurements is displayed from ICU admission up to 72 h. Spearman’s *R* is shown for the individual longitudinal correlation between plasma HBP and NA dose with 72 h together with the *p* value for the analysis.

Abbreviations: *AUD* alcohol use disorder, *C1qD* complement component 1q deficiency, *DM* diabetes mellitus, *CHF* chronic heart failure, *CKD* chronic kidney disease, *CLD* chronic liver disease, *C perf Clostridium perfringens*, *CAI* community-acquired infection, *COPD* chronic obstructive pulmonary disease, *E coli Escherichia coli*, *GI perf* gastrointestinal perforation, *H influenza Haemophilus influenza*, *HAI* hospital-acquired infection, *ICU* intensive care unit, *IE* infective endocarditis, *L pneumo Legionella pneumophila*, *LRI* lower respiratory infection, *MI* myocardial infarction, *MM* multiple myeloma, *NP swab* nasopharyngeal swab, *P micra Parvimonas micra*, *Prevotella spec* Provotella species, *S anginosus Streptococcus anginosus*, *S dysgalactiae Streptococcus dysgalactiae*, *S aureus Staphylococcus aureus*, *S pyogenes Streptococcus pyogenes*, *SOFA* Sequential Organ Failure Assessment, *SSTI* skin and soft tissue infection, *TIA* transient ischemic attack, *UTI* urinary tract infection

### Plasma sample characteristics and kinetics

A total of 370 plasma samples were collected. We excluded 29 samples prior to data analysis due to either of two sources of error: sample haemolysis and delay to lab handling. The 21 samples (6%) with an increased haemolysis index were on average 61% higher in plasma HBP compared to samples with less haemolysis in a generalized mixed-effects model (95% CI 5% to 147%, *p* = 0.03). In the remaining 341 plasma samples, the HBP concentration ranged from 0 to 932 ng/mL (median 47 ng/mL) and PCT ranged from 0 to 83 μg/L (median 31 μg/L). Individual plasma HBP over time can be seen in Fig. 3. In a generalized mixed-effects linear regression model, plasma HBP decreased by an average of 15% per day, with considerable inter- and intraindividual variation and without complete clearance of HBP (95% CI 5% to 25%, *p* = 0.006, *n*_obs_ = 341). Three patients (id 1, 2 and 3) had a clear decrease in HBP over 72 h, averaging 55% decrease per day (95% CI − 62 to − 46%, *p* < 0.001, *n*_obs_ = 38). The remaining 21 patients’ HBP concentrations fluctuated around their individual mean HBP level over time, with a statistically non-significant average decrease of 9% per day (95% CI − 20% to 3%, *p* = 0.14, *n*_obs_ = 303). Plasma HBP sometimes increased to more than 2-fold its previous level and then returned in the next 4-h measurement in these patients, resembling a peak-and-baseline pattern over time. By contrast, individual plasma PCT decreased steadily in practically all patients, averaging a 37% decrease per day in a linear regression model (95% CI − 45 to − 28%, *p* < 0.001, *n*_obs_ = 341). See supplemental figure S1 for individual PCT over time.

### HBP clearance

Because HBP’s mode of clearance is unknown, we explored an association between mean plasma HBP concentrations during the first 3 days of ICU stay and acute or chronic kidney or liver failure, respectively. First, mean HBP was not significantly higher in the 5 patients with chronic kidney disease (CKD) versus the 19 patients without CKD (48 vs 94 ng/ml, *p* = 0.58). Second, individual mean creatinine was not significantly associated with individual mean plasma HBP using Spearman’s *R* (*R* = 0.23, *p* = 0.28). Third, there was also no difference in HBP levels in patients prior to, compared to after, initiation of continuous renal replacement therapy (CRRT) (*p* = 0.29, *n*_pat_ = 7). However, individual mean bilirubin was significantly correlated to mean HBP with an *R* of 0.55 (*p* = 0.006). The two patients (id 10 and 5) with the highest mean HBP also had the highest mean bilirubin in the cohort (HBP 603 and 210 ng/ml, and bilirubin 84 and 78 μmol/L). Only one patient suffered from chronic liver failure (CLF) and his mean HBP was close to the study population mean (65 vs 57 ng/ml).

### HBP and PCT’s association with NA dose over time

**Fig. 2:**
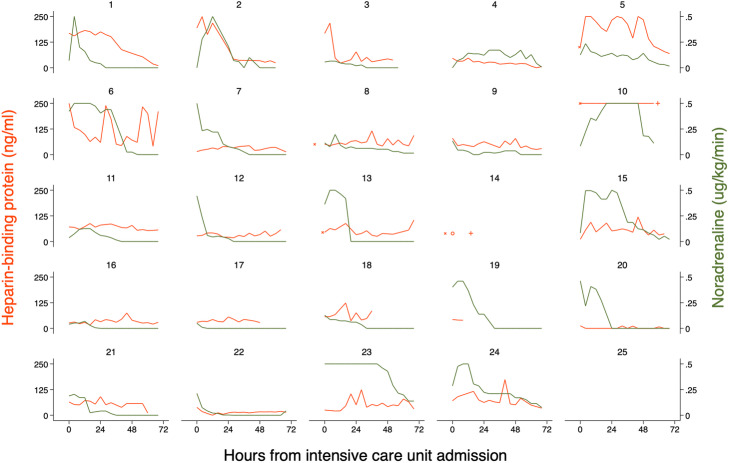
Plasma HBP and NA doses for all 24 patients during the first 72 h of ICU stay. HBP and NA values in this graph are truncated at 250 ng/ml and 0.5 μg/kg/min, respectively. Eight patients are marked with an “*” indicating that they had a significant correlation between plasma HBP and NA dose according to Spearman’s *R*. “x” marks patients’ HBP at the ED, if available. Deaths within 72 h are marked with a “+” (*n* = 2). Patient id 12 only had one HBP measurement in ICU prior to death, marked with an “o”. Patient id 10 is also viewed separately in Suppl. Fig. S2 because of repeated HBP levels above 250 ng/mL

We investigated an association between repeated measures of HBP concentration and NA dose in 24 patients with suspected septic shock within 72 h from ICU admission. In a generalized linear mixed-effects model, every 100 ng/mL increase of HBP was associated with an average 30% increase in NA dose among patients (95% CI 3% to 60%, *p* = 0.03, AIC − 1030). There was considerable variation in the strength of the association between individuals (Fig. 2 and Table 1). Only seven patients had a significant positive correlation according to individual Spearman’s *R* (range 0.56 to 0.74) and one patient had a significant negative *R* of − 0.70. This patient (id 10) had refractory shock and was intentionally weaned off vasopressors before his death at 2.5 days from ICU admission. All patients with a positive correlation were blood culture positive versus only 6 of 15 patients without a correlation (chi-square *p* < 0.01). In addition, correlated patients had a higher mean HBP (96 vs 47 ng/ml, *p* = 0.01) and a higher mean lactate (3.1 vs 2.3 mmol/L, *p* = 0.02) compared to the non-correlated patients. In a corresponding generalized linear mixed-effects model, an increase in 10 μg/L of PCT was significantly correlated to a 74% increase in NA dose among patients (95% CI 54% to 95%, *p* < 0.001, AIC − 1092). NA dose and PCT were significantly correlated in 15 out of 23 patients according to Spearman’s *R*, ranging from 0.65 to 0.99, with no significant negative correlations. Because we analysed repeated measures in the same patient, there was a risk that the correlation between HBP, PCT and NA dose, respectively, was not due to a true effect of the biomarker on the required NA dose, but rather because of a simultaneous decrease over time due to the patient improving. Including time as a confounding co-variate in the model resulted in a loss of the association between NA dose and HBP (95% CI 0.2 to 1.5%, *p* = 0.63), and between NA dose and PCT (95% CI 0.3 to 2.7%, *p* = 0.11), respectively.

### HBPs and PCT’s association with MAP over time

**Fig. 3:**
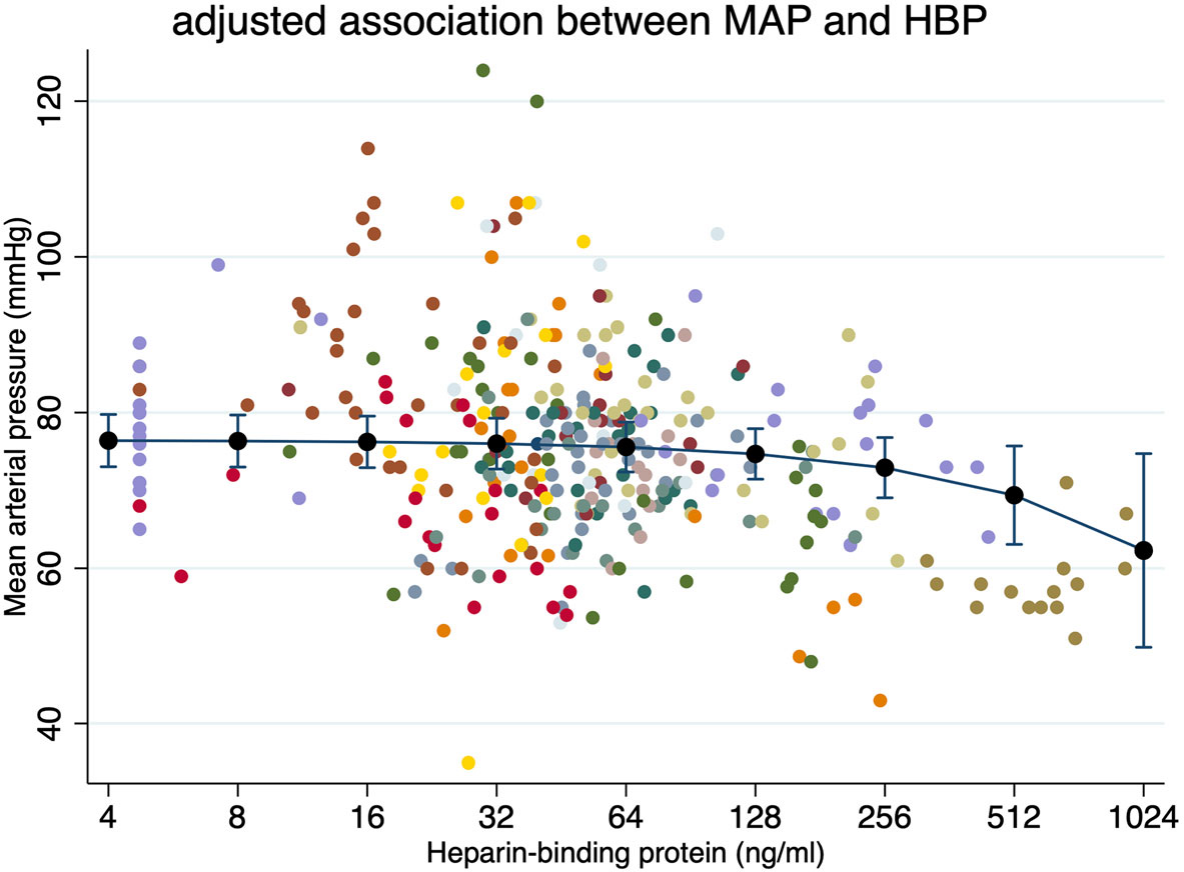
The central black line represents the marginal predicted MAP based on HBP levels in a linear mixed-effects model including noradrenaline dose, vasopressin (yes/no) and time since ICU admission as co-variates. Caps represent 95% confidence intervals for the marginal prediction. Scattered dots are all the actual HBP and MAP values with a separate colour for each patient. HBP is presented on the log scale on the *x*-axis and samples with 0 ng/mL of HBP (*n* = 15) are equalled to the next sample minimum (4.72 ng/mL)

Next we considered MAP as an outcome while adjusting for NA dose and co-administration of vasopressin (yes/no) using linear mixed-effects models. We did this because NA dose may be an inadequate surrogate marker for circulatory failure when other vasopressors are given simultaneously, when NA doses are weaned intentionally or when a desired MAP goal is not reached. In this analysis, HBP was significantly associated to MAP (95% CI − .004 to − 0.3, *p* = 0.01 with level 1 *R*^2^ = 16%) and so was PCT (95% CI − 0.06 to − 0.23, *p* = 0.001 level 1 *R*^2^ = 12%). Including an adjustment for time as a potential confounder removed the association for PCT (95% CI − 0.12 to 0.06, *p* = 0.47) but not for HBP (*p* = 0.04). In the time-adjusted model, every 100 ng/mL increase in HBP corresponded to a 1.4-mmHg decrease in MAP (95% CI − 1 to − 2.3 mmHg, level 1 *R*^2^ = 18%) (see Fig. 3). A likelihood ratio test confirmed the better fit for the HBP model with time included (*p* < 0.001). To further investigate support for a causal relationship between HBP and MAP in our data, we entered all potential confounders based on previous knowledge in a full linear mixed-effects model including the following co-variates: age, gender, chronic heart failure (yes/no), SAPS 3 score, SOFA score, lactate, time since ICU admission, NA dose and vasopressin (yes/no). HBP was not significantly associated with MAP in the full model (95% CI − 0.02 to 0.02, *p* = 0.91) and neither was PCT in a corresponding model (95% CI − 0.15 to 0.02, *p* = 0.14). However, their estimated ß-coefficient remained in the hypothesized direction (− 0.001 for HBP and − 0.06 for PCT).

### HBPs and PCT’s association with SVRI over time

Data on SVRI and CI was available from non-invasive monitoring in 13 patients (9 on PiCCO and 4 on NICOM). Plasma HBP was not associated with SVRI over time in a crude linear mixed-effects model or in a model adjusting for time only (*p* = 0.09 and *p* = 0.21) and neither was PCT in two corresponding models (*p* = 0.33 and *p* = 0.08). However, every 1 ng/mL increase in HBP was associated with 1 dyne s cm^−5^ m^−2^ decrease in SVRI in a model adjusting for time, CI and NA dose (95% CI − 0.36 to − 1.62, *p* = 0.002, *n*_obs_ = 111, level 1 *R*^2^ = 68%, suppl. Fig. S3) which was not true for a corresponding model on PCT (*p* = 0.73). A likelihood ratio test confirmed the better fit of the HBP to SVRI model with NA and CI included compared to time alone (*p* < 0.001) but did not support a significantly better fit for a larger model also including vasopressin dose, dobutamine dose, heart rate and levosimendan (yes/no) as added co-variates (*p* = 0.26 and level 1 *R*^2^ = 70%). The ß-coefficient and *p* value for HBP remained largely the same in the smaller and the larger model (coef. − 0.88 and *p* = 0.009) and PCT still lacked an association with SVRI in a corresponding larger model (*p* = 0.94).

### Association between total HBP and PCT exposure and total NA need

To explore HBP’s association with NA dose beyond temporary variations, we investigated if the overall HBP exposure during the 72-h study period was associated with the total noradrenaline requirement. To do this, we calculated an area under curve (AUC) for HBP, PCT and NA dose, respectively for each patient, and evaluated the overall association between biomarker AUC and NA AUC using Spearman’s *R*. The AUC for HBP was significantly correlated to the AUC for NA (*R* = 0.49, *p* = 0.04) while the AUC for PCT was not in a corresponding analysis (*R* = 0.35. *p* = 0.10).

### HBP levels and sepsis severity

In a linear mixed-effects regression model, a higher daily individual mean HBP was associated with a higher SOFA score in the first 3 days of ICU stay (95% CI 0.003 to 0.023, *p* = 0.01, level 1 *R*^2^ = 15%). The association was also significant between mean daily PCT and daily SOFA score but with a lower *R*^2^ (95% CI 0.03 to 0.12, *p* = 0.001, level 1 *R*^2^ = 2%). There was also a significant correlation between mean HBP, mean platelet count and mean lactate levels during the first 72 h, respectively (*R* = − 0.47, *p* = 0.02 and *R* = 0.58, *p* = 0.003). The correlations between mean PCT and mean platelets, and mean PCT and mean lactate were not statistically significant (*R* = 0.06, *p* = 0.77 and *R* = 0.12, *p* = 0.58).

## Discussion

### Key findings

We have found an overall significant association between serial plasma heparin-binding protein concentration and cardiovascular organ dysfunction severity during the first 3 days of ICU stay in patients with septic shock. The association was stronger for HBP than for PCT in most analyses. However, the association between HBP and MAP or NA dose was not as strong or uniform as we had hypothesized and repeated measures of HBP showed an unexpected and previously undescribed peak-and-baseline pattern over time with large variation within and between patients.

### HBP association to cardiovascular organ dysfunction and systemic vascular resistance

The aim of the study was to see if the degree of circulatory failure in septic shock would be linked to the concentration of HBP in plasma. Experimental studies have demonstrated that HBP induces cytoskeletal rearrangement and loosening of tight junctions in the endothelium. This enables neutrophil migration to the infected tissue but also leads to vascular leak and endothelial dysfunction [2, 3, 8]. We found that every 100 ng/mL increase in HBP over time corresponded to a 30% increase in NA dose in a crude analysis or a 1.4-mmHg decrease in MAP in an adjusted analysis. Furthermore, the total HBP exposure during the first 3 days of ICU stay had a relatively strong correlation to the total NA dose administered (*R* = 0.49). These findings support our overall hypothesis that plasma HBP concentrations over time are associated with cardiovascular organ dysfunction severity. We also found 1 dyne s cm^−5^ m^−2^ decrease in SVRI for every 1 ng/mL increase in HBP when adjusting for time and cardiac output index in a subgroup of patients with non-invasive monitoring. This exploratory analysis could support the idea that the driving factor behind HBP association with cardiovascular organ dysfunction is mainly through a decrease in systemic vascular resistance, rather than cardiac contractility.

### Strength of the association for HBP compared to PCT

Procalcitonin was equally or more closely correlated to NA dose compared to HBP in several initial crude analyses including individual Spearman’s *R* and a generalized mixed-effects model. This was unexpected since PCT does not have an equally well-established physiological connection to endothelial dysfunction. However, a possible link between PCT and nitric oxide increase has been suggested [19]. Possibly, the strength of the association is explained by PCT’s steady decrease in practically all patients in the first 72 h from ICU admission (Fig. S1), during which time most patients get clinically better. HBP was much more variable over time, weakening the overall association. A subsequent analysis including an adjustment for time since ICU admission as a possible confounder removed the association for PCT and MAP or NA dose, respectively, contradicting a causative role. In contrast, an increase in HBP was still associated to a decrease in MAP when adjusting for time and vasopressors. Still, we could not find support for a causative role of HBP on MAP when adjusting for possible confounders in our data. PCT was not associated with SVRI in any of the tested exploratory models, in contrast to HBP.

### Individual variation in the strength of the association and a “HBP-positive δ-like phenotype”

There was considerable variation in the strength of the association between cardiovascular organ dysfunction and HBP between individuals. This may reflect the heterogenous study population. Patients with a significant HBP to NA dose correlation were more often blood culture positive, had higher mean lactate levels and had higher HBP levels. It was also the highest HBP values (> 150 ng/mL) that were most clearly associated with a concurrent decrease in MAP and SVRI, conceivably driving much of the overall effect (see Fig. 3 and S3). This raises the question if there is a threshold at which HBP concentrations exhibit a clinical effect on systemic vascular resistance. In recent experimental studies, plasma concentrations of 10,000 ng/mL were used in vitro to study the effects of HBP on renal cells [20] and concentrations above 50,000 ng/mL were used in vivo to evaluate HBP’s effect on lung tissue in mice [8]. In our study, median HBP was 47 ng/mL and only about 10% of samples was above 200 ng/mL, similar to the levels reported in septic shock patients by Linder et al. in 2012 (median 30 ng/mL and maximum 1277 ng/mL). Perhaps it is unreasonable to expect a detectable physiological effect at the lower HBP levels we are measuring in plasma. We also saw that patients with higher mean daily HBP had a higher daily SOFA score and that mean HBP levels were correlated to a lower platelet count, higher mean bilirubin levels and higher mean lactate levels. This is in line with earlier studies from the ICU that have shown higher mortality from sepsis in patients with higher HBP levels [12, 21]. Intriguingly, these findings may indicate the existence of a “HBP-positive” septic shock phenotype that is driven by strong neutrophil activation, liver failure and circulatory failure and seem to resemble the delta (δ)-subtype described by Seymour et al. [22].

### A peak-and-baseline pattern for HBP over time

In contrast to our expectations, based on other inflammatory biomarkers such as PCT and C-reactive protein, plasma HBP did not display a steady decrease over time, but rather a kind of peak-and-baseline kinetic pattern. Considering that HBP is an inducer of vascular permeability, both enabling neutrophils’ extravasation and augmenting vascular leak, it would reasonably be most favourable to the host and the neutrophil if the effect of HBP was strong but short, minimizing the adverse effects. The circulating half-life of neutrophils may be as brief as 6 to 8 h [23] and up to 90% of their HBP from secretory vesicles have been shown to be released within 30 min of activation [3]. There is also data from mice where over 99% of recombinant HBP was cleared from the blood 1 h after a high-dose injection (from conc. ~ 100,000 to ~400 ng/mL) [20]. Whether this is an effect of distribution or elimination left aside, these findings can conform with the short-lived peaks observed in many of our patients, and we cannot rule out that there are even more peaks within the 4-h sampling periods used in this study. Our finding of repeated HBP peaks within 72 h contrasts a study of 10 patients with burns who had a uniform steady decrease in daily plasma HBP concentration over days 1–3 from admission [24]. Patients in our cohort also did not completely clear their HBP within 72 h. There is data indicating that an elevated plasma HBP is associated to acute kidney injury in sepsis [20, 21], but if this is because of a direct effect of HBP on the kidney or if it is due to an accumulation from decreased filtration is currently unknown. A recent study found no support for HBP clearance by the kidney or by CRRT [25]. This is in line with our data which showed no association between HBP and CKD, longitudinal creatinine or initiation of CRRT, respectively. Instead we found a correlation between mean bilirubin levels and mean HBP levels which may serve as a clue that liver function may be involved. Liver disease is a well-established risk factor in the critically ill with infection [26] and an interaction between HBP, liver failure and disease severity pose an interesting concept for further study. Lastly, the kinetic pattern of HBP described in this study with short-lived peaks and incomplete clearance could have implications for the interpretation of previous single-sample HBP studies. Whether you identify a patient’s peak or baseline HBP may directly affect the biomarkers estimated prognostic accuracy. It may also have implications for the design of future plasma HBP studies, which may consider repeated sampling.

### Limitations

There are some important limitations of this study. First, the study lacks a sample size calculation and a pre-specified analysis plan. This was difficult to achieve since no comparable studies have been conducted and the study should be considered exploratory. Second, patients were included during two separate study periods at two different centres because of local inclusion difficulties at the first centre and only 25% of eligible patients with septic shock were included at the second centre. This may have led to selection bias and impair generalisability. However, baseline characteristics for non-included patients resemble the final study cohort. Third, studying a limited number of patients during a relatively short study period resulted in low power in some exploratory analyses, few patients with treatment failure and later clinical events of importance may have been missed. Fourth, some data was collected retrospectively from charts which makes timely connections to clinical events difficult. Fifth, the outcome measures of MAP, NA dose and SVRI are only surrogate markers of the true physiological situation. Strengths of the study are the prospective design, wide inclusion criteria and a well-characterized cohort in which 23 out of 24 patients had a final diagnosis of septic shock. The frequent plasma sampling, a standardized biomarker-analysis and a contemporary statistical approach to handling of repeated measures were additional strengths.

## Conclusions

Plasma HBP displayed a previously undescribed peak-and-baseline pattern over time. Repeated measures of HBP concentration were associated to cardiovascular organ dysfunction severity during septic shock. This was most pronounced in patients with the highest HBP levels and the highest disease severity.

## Supplementary information

**Additional file 1: Figure S1.** Procalcitonin and NA doses for all 24 patients during the first 72 hours of ICU stay. NA values are truncated at 0.5 μg/kg/min. Deaths within 72 hours are marked with a “+” (n = 2). Patient id 12 only had one HBP measurement in ICU prior to death, marked with an “o”. **Figure S2.** Plasma HBP and NA doses for patient number 10 during the first 72 hours of ICU stay. This patient’s graph is shown separately because his high HBP levels were truncated in Fig. 2. The patient died due to refractory septic shock at 59 hours from ICU admission marked with an “+”. **Figure S3.** The central black line represents the marginal predicted SVRI based on a linear mixed-effects model including HBP levels, NA dose time and CI as co-variates among 13 patients with non-invasive monitoring. Caps represent 95% confidence intervals for the marginal prediction. Scattered dots are all the actual SVRI and HBP values with a separate colour for each patient. HBP is presented on the log scale on the x-axis and samples with 0 ng/mL of HBP are equalled to the next sample minimum (4.72 ng/mL).

**Additional file 2.** STROBE checklist.

**Additional file 3.** Stata code.

## Data Availability

The datasets used and/or analysed in the current study are available from the corresponding author on reasonable request.

## Abbreviations

HBP: Heparin-binding protein
ICU: Intensive care unit
SOFA: Sequential Organ Failure Assessment
PCT: Procalcitonin
ED: Emergency department
ELISA: Enzyme-linked immunosorbent assay
MAP: Mean arterial pressure
NA: Noradrenaline
CCI: Charlson comorbidity index
AUC: Area under the curve
AIC: Akaike information criterion
*R*^2^: Coefficient for determination
SAPS 3: Simplified acute physiology score 3
IBD: Inflammatory bowel disease
C1q: Complement component 1q
CRRT: Continuous renal replacement therapy
CHF: Chronic heart failure
SVRI: Systemic vascular resistance index
CI: Cardiac index
PiCCO: Pulse contour cardiac output
NICOM: Non-invasive cardiac output monitoring
δ: Delta (Greek)
