# Supplementary figures and images for: Repeated measures of Heparin-binding protein (HBP) and procalcitonin during septic shock: biomarker kinetics and association with cardiovascular organ dysfunction
Source: Intensive Care Med Exp. 2020 Sep 10;8:51. doi: 10.1186/s40635-020-00338-8 (PMC7483682; doi:10.1186/s40635-020-00338-8)

Procalcitonin (ug/ml)

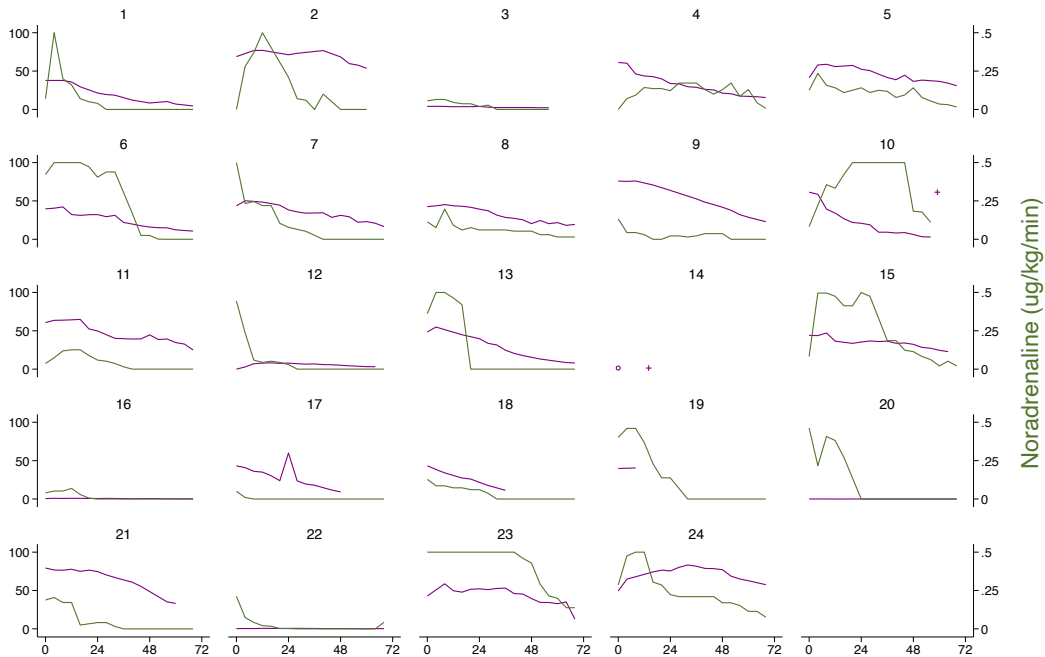

Noradrenaline (ug/kg/min)

Hours from intensive care unit admission

Supplement: Supplementary file 1 — Additional file 1: Figure S1. Procalcitonin and NA doses for all 24 patients during the first 72 hours of ICU stay. NA values are truncated at 0.5 μg/kg/min. Deaths within 72 hours are marked with a “+” (n = 2). Patient id 12 only had one HBP measurement in ICU prior to death, marked with an “o”. Figure S2. Plasma HBP and NA doses for patient number 10 during the first 72 hours of ICU stay. This patient’s graph is shown separately because his high HBP levels were truncated in Fig. 2. The patient died due to refractory septic shock at 59 hours from ICU admission marked with an “+”. Figure S3. The central black line represents the marginal predicted SVRI based on a linear mixed-effects model including HBP levels, NA dose time and CI as co-variates among 13 patients with non-invasive monitoring. Caps represent 95% confidence intervals for the marginal prediction. Scattered dots are all the actual SVRI and HBP values with a separate colour for each patient. HBP is presented on the log scale on the x-axis and samples with 0 ng/mL of HBP are equalled to the next sample minimum (4.72 ng/mL). [file 40635_2020_338_MOESM1_ESM.zip › fig_s1_tverring.pdf]

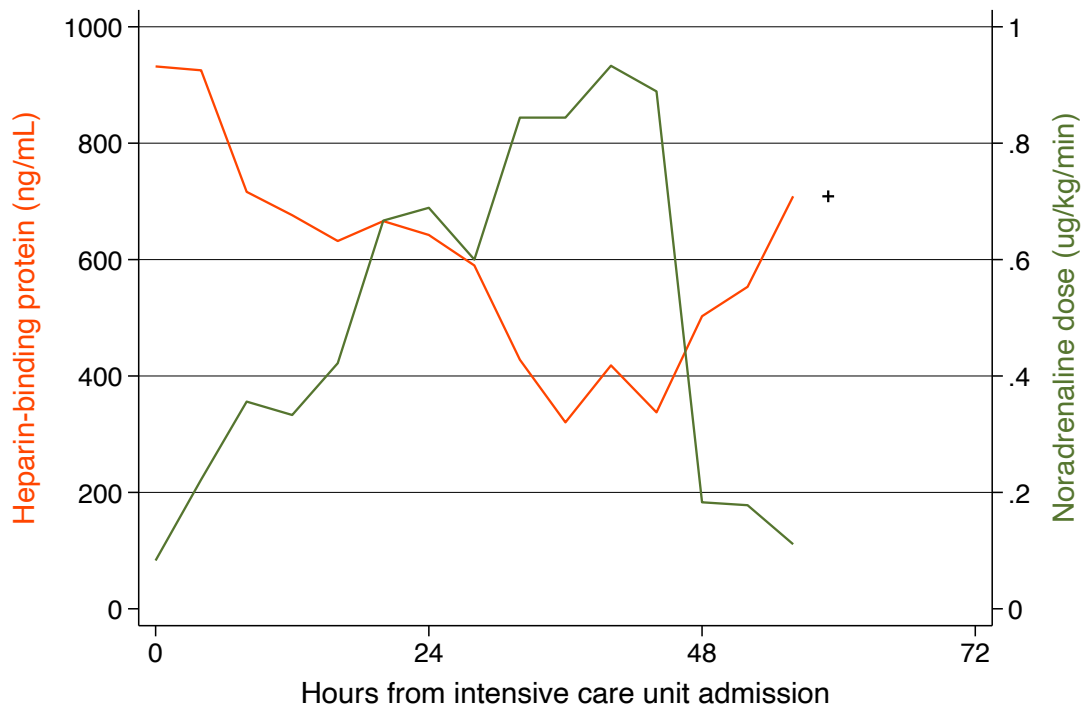

Supplement: Supplementary file 1 — Additional file 1: Figure S1. Procalcitonin and NA doses for all 24 patients during the first 72 hours of ICU stay. NA values are truncated at 0.5 μg/kg/min. Deaths within 72 hours are marked with a “+” (n = 2). Patient id 12 only had one HBP measurement in ICU prior to death, marked with an “o”. Figure S2. Plasma HBP and NA doses for patient number 10 during the first 72 hours of ICU stay. This patient’s graph is shown separately because his high HBP levels were truncated in Fig. 2. The patient died due to refractory septic shock at 59 hours from ICU admission marked with an “+”. Figure S3. The central black line represents the marginal predicted SVRI based on a linear mixed-effects model including HBP levels, NA dose time and CI as co-variates among 13 patients with non-invasive monitoring. Caps represent 95% confidence intervals for the marginal prediction. Scattered dots are all the actual SVRI and HBP values with a separate colour for each patient. HBP is presented on the log scale on the x-axis and samples with 0 ng/mL of HBP are equalled to the next sample minimum (4.72 ng/mL). [file 40635_2020_338_MOESM1_ESM.zip › fig_s2_tverring.pdf]

# adjusted association between SVRI and HBP

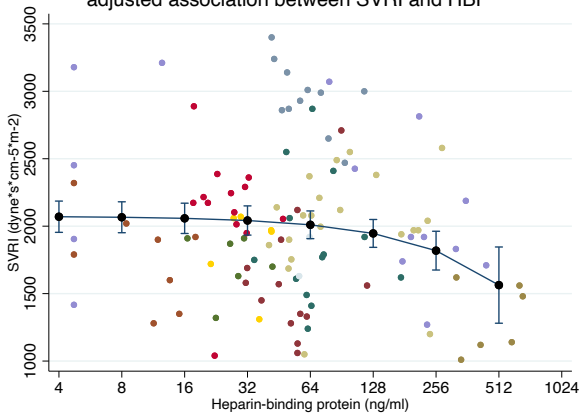

Supplement: Supplementary file 1 — Additional file 1: Figure S1. Procalcitonin and NA doses for all 24 patients during the first 72 hours of ICU stay. NA values are truncated at 0.5 μg/kg/min. Deaths within 72 hours are marked with a “+” (n = 2). Patient id 12 only had one HBP measurement in ICU prior to death, marked with an “o”. Figure S2. Plasma HBP and NA doses for patient number 10 during the first 72 hours of ICU stay. This patient’s graph is shown separately because his high HBP levels were truncated in Fig. 2. The patient died due to refractory septic shock at 59 hours from ICU admission marked with an “+”. Figure S3. The central black line represents the marginal predicted SVRI based on a linear mixed-effects model including HBP levels, NA dose time and CI as co-variates among 13 patients with non-invasive monitoring. Caps represent 95% confidence intervals for the marginal prediction. Scattered dots are all the actual SVRI and HBP values with a separate colour for each patient. HBP is presented on the log scale on the x-axis and samples with 0 ng/mL of HBP are equalled to the next sample minimum (4.72 ng/mL). [file 40635_2020_338_MOESM1_ESM.zip › fig_s3_tverring.pdf]
