# Supplementary material for: Repeated measures of Heparin-binding protein (HBP) and procalcitonin during septic shock: biomarker kinetics and association with cardiovascular organ dysfunction
Source: Intensive Care Med Exp. 2020 Sep 10;8:51. doi: 10.1186/s40635-020-00338-8 (PMC7483682; doi:10.1186/s40635-020-00338-8)
Supplement: Supplementary file 3 — Additional file 3. Stata code. [file 40635_2020_338_MOESM3_ESM.pdf]

```
/// *** Stata code for Tverring et al manuscript *** ///

*** Version: Stata/MP 16.0 for Mac (64-bit Intel) ***

** Date: 20april2020 **

// Analyses are reported in the order the results appear in
the manuscript //

*** Explanation to variables in dataset:

* id = patient id
* hbp = heparin-binding protein (ng/ml) (n=370)
* hbpx = hbp + 0.001 (to avoid zero values in dependent
variable of the gamma-distribution analyses)
* hbphl = a hbp variable where all variables with hemolysis
and lag-time at lab has been excluded (n=341)
* hbphlx = hbphl + 0.001
* hbphld = mean daily hbphl from day 1 to 3
* hbphlx4 = hbphl where zero-values (n=15) have been equaled
to the next sample minimum (4.72 ng/mL) to allow graphical
display on the log-scale
* pct = procalcitonin (µg/l)
* pcthl = pct-variable with increased hemolysis and lag-time
excluded
* pcthlx = pcthl + 0.001
* hemolys01 = a dichotomized hemolysis-variable at or above 10
(1/0)
* cread = daily creatinine (µmol/L)
* bilid = daily bilirubin (µmol/L)
* crrthbp01 = dichotomized variable to indicate ongoing
continous renal replacement therapy (CRRT) or not (1/0)
* ccickd = chronic kidney disease (CKD) classified according
to charlson comorbidity index (CCI)
* na = noradrenaline dose (µg/kg/min)
* nax = na + 0.001
* bcpos = blood culture positive (1/0)
* signcorr = patients with versus without a singificant
positive HBP-to-NA correlation (1: n=7 and 0: n=15)
* time = time elapsed since ICU admission (hours)
* vasopressin01 = administration of vasopressin (1/0)
* lactate = lactate (mmol/L, continous)
* sofa = daily sequential organ failure assessment (continous)
* sapsiii = simplified acute physiology score III at ICU
admission (cont.)
* age = age from birth to study inclusion (years, cont)
* female = female gender (1/0)
* auchbphl = individual HBP area under the curve (AUC)
calculated using the pkexamine-command
* aucpcthl = individual PCT AUC
* svri = systemic vascular resistance index (dyne*s*cm-5*m-2)
```

```
* ci = cardiac index (l/min/m2)
* puls = heart rate (cont.)
* levosimendan = administration of levosimendan (1/0)
* dobutamin01 = administration of dobutamin (1/0)

* - Dataset is in long format

* - Comminuty-contributed Ado packages used
* qgamma (Multi-level tools)
* mlt (Quantile-quantile plot for data versus fitted gamma
distribution)

// ** Do-file ** //

use "/Users/jonastverring/Desktop/HBP
KISS/stata_dta/hbpkissdata_16april2020.dta"

* Difference in HBP between samples with vs without an
increased hemolysis-index
meglm hbpx hemolys01 || id: , family(gamma) eform

* HBP and PCT change per day using generelized linear mixed
models, gamma distrubution and a random slope for time
meglm hbphlx timed || id: timed , family(gamma) eform
meglm hbphlx timed if id<=3 || id: timed , family(gamma) eform
meglm hbphlx timed if id>3 || id: timed , family(gamma) eform
meglm pcthlx timed || id: timed , family(gamma) eform

* Overall mean HBP levels in patients with vs without chronic
kidney injury
ttest meanhbphlbyid if nr==1, by(ccickd)
ranksum meanhbphlbyid if nr==1, by(ccickd)

* Overall mean HBP levels and correlation with mean creatine
and mean bilirubin within 72 hours respectively
spearman meanhbphlbyid meancreabyid if nr==1
spearman meanhbphlbyid meanbilibyid if nr==1

* HBP levels over time in patient before versus after
initiation of continous renal replacement therapy.
meglm hbphlx i.crrthbp01 || id: , family(gamma) eform

* Overall correlation between NA dose and HBP and model fit
according to Akaike's information criterion (AIC)
meglm nax hbphl || id: , family(gamma) eform
estat ic

* Individual spearman's R for correlation between NA dose and
HBP
bys id: spearman na hbphl
```

```
* Difference in clinical characteristics of patient with
versus without a significant positive HBP-to-NA correlation
tab bcpos signcorr if nr==1, chi col
ranksum meanhbphlid if nr==1, by(signcorr)
ranksum meanlactid if nr==1, by(signcorr)

* Overall correlation between NA dose and PCT and model fit by
AIC
meglm nax pcthl || id: , family(gamma) eform
estat ic

* Individual correlation between NA dose and PCT
bys id: spearman na pcthl

* Correlation between NA dose and HBP including an adjustment
for time and a random slope
meglm nax hbphl time || id: time, family(gamma) eform

* Correlation between NA dose and PCT including time
meglm nax pcthl time || id: time, family(gamma) eform

* Assumptions for meglm and family(gamma), applied to all
meglm-models above
predict pres, pearson
qgamma pres, name(qgpres, replace)
drop pres

* HBPs correlation to MAP when adjusting for NA dose and
vasopressin (yes/no)) and calculation of R2
xtmixed map hbphl na vasopressin01 || id:
mltrsq
est store A

* PCTs correlation to MAP when adjusting vasopressors
xtmixed map pcthl na vasopressin01 || id:
mltrsq

* PCTs correlation to MAP adjusting for vasopressors and time
with a random slope.
xtmixed map pcthl na vasopressin01 time || id: time, cov(un)

* HBPs correlation to MAP adjusting for vasopressors and time
xtmixed map hbphl na vasopressin01 time || id: time, cov(un)
mltrsq
est store B

* Likelihood ratio test between nested MAP to HBP models with
and without time
lrtest A B
```

```
* Fully adjusted model exploring HBPs potential causal link to
MAP
xtmixed map hbphl time lactate sofa sapsiii age female ccichf
vasopressin01 na || id: time, cov(un)
mltrsq

* Corresponding full model of PCT on MAP
xtmixed map pcthl time lactate sofa sapsiii age female ccichf
vasopressin01 na || id: time, cov(un)

* Checking for multicollinearity
quietly regress map hbphl time lactate sofa sapsiii age female
ccichf vasopressin01 na
estat vif

* HBPs association to SVRI, first a crude model and then with
time as a co-variate and random slope
xtmixed svri hbphl || id:
xtmixed svri hbphl time || id: time, cov(un)

* HBPs association to SVRI, small model versus time only with
a likelihood ratio test for model fit
xtmixed svri hbphl time na ci || id: time, cov(un)
est store A
mltrsq
xtmixed svri hbphl time if ci!=. & na!=. || id: time, cov(un)
est store B
lrtest A B

* HBPs association to SVRI, small model with time, NA dose and
CI and larger model and a LR test between the two models
xtmixed svri hbphl time na ci || id: time, cov(un)
est store C
xtmixed svri hbphl time na vasopressin01 dobutamin01 ci puls
levosimendan || id: time, cov(un)
est store D
mltrsq
lrtest C D

* Checking for multicollinearity
quietly regress svri hbphl time na vasopressin01 dobutamin01
ci puls levosimendan
estat vif

* Corresponding models on PCTs crude and adjusted associations
to SVRI
xtmixed svri pcthl || id:
xtmixed svri pcthl time || id: time, cov(un)
xtmixed svri pcthl time na ci || id: time, cov(un)
xtmixed svri pcthl time na vasopressin01 dobutamin01 ci puls
levosimendan || id: time, cov(un)
```

```
* Assumptions for xtmixed, applied to all xtmixed-models
above.
predict fitted, fitted
predict residual, residual
twoway (scatter residual fitted) (lfit residual fitted),
name(twfit, replace)
qnorm residual, name(qnormfit, replace)
drop fitted residual

* Correlation between total "HBP exposure" and NA dose
spearman aucna auchbphl if nr==1

* Correlation between total "PCT exposure" and NA dose
spearman aucna aucpcthl if nr==1

* Correlatiom between mean daily HBP and daily SOFA during day
1-3, with R2
xtmixed sofad hbphld || id:
mltrsq

* Correlatiom between mean daily HBP and daily SOFA
xtmixed sofad pcthld || id:
mltrsq

*Correlation between mean HBP within 72 hours and mean
thrombocyte count and mean lactate levels respectively
spearman meanhbphlbyid meantrcbyid if nr==1
spearman meanhbphlbyid meanlactbyid if nr==1

*Corresponding correlation for PCT and trc and lactate
spearman meanpcthlbyid meantrcbyid if nr==1
spearman meanpcthlbyid meanlactbyid if nr==1

// Figures

* Fig 3: Individual HBP and NA dose over time
twoway line hbpmax250 time if time<72 & hemolys<10 &
lagtimelab<=60 | time<72 & hemolys==. & lagtimelab==.,
lcolor(orange_red) yaxis(1) ytitle( "Heparin-binding protein
(ng/ml)", size(*.8) color(orange_red) axis(1)) xlabel(0 24 48
72) ylabel(0 125 250, nogrid axis(1)) xtitle("Hours from
intensive care unit admission", size(*.8)) by(id, legend(off)
note("")) || line naxx5 time if time<72, yaxis(2) ylabel(0
0.25 0.5, axis(2)) by(id, r1title("Noradrenaline (ug/kg/min)",
color( forest_green) size(*.8)))
name(hbpmax250andnamax05color, replace) lstyle(solid) lcolor(
forest_green) || scatter hbpx time if time<72 & hemolys<=10 &
lagtimelab<=60 & id==14, msymbol(oh) mcolor(orange_red) ||
scatter hbped250 timehbped if nr==1, msymbol(x)
mcolor(orange_red) || scatter meanhbpid250 icutodeath if
```

```
icutodeath<72 & icutodeath!=. & nr==1, msymbol(+)
mcolor(orange_red)

* Fig 4: Association between MAP and HBP adjusted for NA dose,
vasopressin (yes/no) and time
xtmixed map hbphlx4 na vasopressin01 time || id: time, cov(un)
margins, at(hbphlx4=(4 8 16 32 64 128 256 512 1024))
marginsplot , name(assochbpxtmixed, replace) legend(off)
scheme(s2gcolor) graphregion(fcolor(white))
plotopts(mcolor(black)) ytitle("Mean arterial pressure
(mmHg)", size(*.9)) xtitle("Heparin-binding protein (ng/ml)",
size(*.9)) title("adjusted association between MAP and HBP" ,
color(black)) addplot((scatter map hbphlx4 if id==1, below
xlabel(4 8 16 32 64 128 256 512 1024) xscale(log)
msymbol(smcircle)) (scatter map hbphlx4 if id==2,
msymbol(smcircle)) (scatter map hbphlx4 if id==3,
msymbol(smcircle)) (scatter map hbphlx4 if id==4,
msymbol(smcircle)) (scatter map hbphlx4 if id==5,
msymbol(smcircle)) (scatter map hbphlx4 if id==6,
msymbol(smcircle)) (scatter map hbphlx4 if id==7,
msymbol(smcircle)) (scatter map hbphlx4 if id==8,
msymbol(smcircle)) (scatter map hbphlx4 if id==9,
msymbol(smcircle)) (scatter map hbphlx4 if id==10,
msymbol(smcircle)) (scatter map hbphlx4 if id==11,
msymbol(smcircle)) (scatter map hbphlx4 if id==12,
msymbol(smcircle)) (scatter map hbphlx4 if id==13,
msymbol(smcircle)) (scatter map hbphlx4 if id==14,
msymbol(smcircle)) (scatter map hbphlx4 if id==15,
msymbol(smcircle)) (scatter map hbphlx4 if id==16,
msymbol(smcircle)) (scatter map hbphlx4 if id==17,
msymbol(smcircle)) (scatter map hbphlx4 if id==18,
msymbol(smcircle)) (scatter map hbphlx4 if id==19,
msymbol(smcircle)) (scatter map hbphlx4 if id==20,
msymbol(smcircle)) (scatter map hbphlx4 if id==21,
msymbol(smcircle)) (scatter map hbphlx4 if id==22,
msymbol(smcircle)) (scatter map hbphlx4 if id==23,
msymbol(smcircle)) (scatter map hbphlx4 if id==24,
msymbol(smcircle)))

* Fig S1: Individual PCT and NA dose over time
twoway line pcthl time if time<72 & hemolys<10 &
lagtimelab<=60 | time<72 & hemolys==. & lagtimelab==.,
lcolor(purple) yaxis(1) ytitle( "Procalcitonin (ug/ml)",
size(*.8) color(purple) axis(1)) xlabel(0 24 48 72) ylabel(0
50 100, nogrid axis(1)) xtitle("Hours from intensive care unit
admission", size(*.8)) by(id, legend(off) note("")) || line
naxx5 time if time<72, yaxis(2) ylabel(0 0.25 0.5, axis(2))
by(id, r1title("Noradrenaline (ug/kg/min)",
color(forest_green) size(*.8))) name(pcthlandnamax05color,
replace) lstyle(solid) lcolor(forest_green) || scatter pcthl
time if id==14, msymbol(oh) mcolor(purple)  || scatter pcthl
```

```
icutodeath if icutodeath<72 & icutodeath!=. & nr==1,
msymbol(+) mcolor(purple)

* Fig S2: HBP over time for patient id 10
twoway line hbphl time if id==10, lcolor(orange_red)
legend(off) yaxis(1) ytitle( "Heparin-binding protein
(ng/ml)", size(*.8) color(orange_red) axis(1)) xlabel(0 24 48
72) ylabel(0 200 400 600 800 1000) xtitle("Hours from
intensive care unit admission", size(*1)) note("") || line na
time if id==10, yaxis(2) lstyle(solid) lcolor(forest_green)
ytitle("Noradrenaline (ug/kg/min)", axis(2)
color(forest_green)) || scatter hbphlcopy icutodeath if id==10
& nr==15, msymbol(+) mcolor(black)

* Fig S3: Association between SVRI and HBP adjusted for
cardiac index, NA dose and time
xtmixed svri hbphlx4 time na ci || id: time, cov(un)
margins, at(hbphlx4=(4 8 16 32 64 128 256 512))
marginsplot , name(assochbpsvrixtmixed, replace) legend(off)
scheme(s2gcolor) graphregion(fcolor(white))
plotopts(mcolor(black)) ytitle("SVRI (dyne*s*cm-5*m-2)",
size(*.9)) xtitle("Heparin-binding protein (ng/ml)",
size(*.9)) title("adjusted association between SVRI and HBP",
color(black)) addplot((scatter svri hbphlx4 if id==1, below
xlabel(4 8 16 32 64 128 256 512 1024) xscale(log)
msymbol(smcircle)) (scatter svri hbphlx4 if id==2,
msymbol(smcircle)) (scatter svri hbphlx4 if id==3,
msymbol(smcircle)) (scatter svri hbphlx4 if id==4,
msymbol(smcircle)) (scatter svri hbphlx4 if id==5,
msymbol(smcircle)) (scatter svri hbphlx4 if id==6,
msymbol(smcircle)) (scatter svri hbphlx4 if id==7,
msymbol(smcircle)) (scatter svri hbphlx4 if id==8,
msymbol(smcircle)) (scatter svri hbphlx4 if id==9,
msymbol(smcircle)) (scatter svri hbphlx4 if id==10,
msymbol(smcircle)) (scatter svri hbphlx4 if id==11,
msymbol(smcircle)) (scatter svri hbphlx4 if id==12,
msymbol(smcircle)) (scatter svri hbphlx4 if id==13,
msymbol(smcircle)) (scatter svri hbphlx4 if id==14,
msymbol(smcircle)) (scatter svri hbphlx4 if id==15,
msymbol(smcircle)) (scatter svri hbphlx4 if id==16,
msymbol(smcircle)) (scatter svri hbphlx4 if id==17,
msymbol(smcircle)) (scatter svri hbphlx4 if id==18,
msymbol(smcircle)) (scatter svri hbphlx4 if id==19,
msymbol(smcircle)) (scatter svri hbphlx4 if id==20,
msymbol(smcircle)) (scatter svri hbphlx4 if id==21,
msymbol(smcircle)) (scatter svri hbphlx4 if id==22,
msymbol(smcircle)) (scatter svri hbphlx4 if id==23,
msymbol(smcircle)) (scatter svri hbphlx4 if id==24,
msymbol(smcircle)))

/// *** End of Do-file *** ///
```
